# An Investigation of Associations Between Management and Feather Damage in Canadian Laying Hens Housed in Furnished Cages

**DOI:** 10.3390/ani9040135

**Published:** 2019-03-31

**Authors:** Caitlin Decina, Olaf Berke, Nienke van Staaveren, Christine F. Baes, Tina M. Widowski, Alexandra Harlander-Matauschek

**Affiliations:** 1 Department of Population Medicine, Ontario Veterinary College, University of Guelph, Guelph, ON N1G 2W1, Canada; cdecina@uoguelph.ca (C.D.); oberke@uoguelph.ca (O.B.); 2 Department of Animal Biosciences, Ontario Agricultural College, University of Guelph, Guelph, ON N1G 2W1, Canada; nvanstaa@uoguelph.ca (N.v.S.); cbaes@uoguelph.ca (C.F.B.); twidowsk@uoguelph.ca (T.M.W.)

**Keywords:** chickens, feather pecking, furnished cage, welfare

## Abstract

**Simple Summary:**

Feather damage due to feather pecking behaviour remains a serious welfare concern in flocks of egg-laying hens housed in large groups. A better understanding of the farm factors that contribute to feather damage is needed, especially as Canadian egg farming transitions away from conventional cage housing systems and into alternative, larger group systems. This study aimed to explore bird, housing, and management associations with feather damage in Canadian laying hens housed in furnished cage systems. Twenty-six laying hen farms housing birds in furnished cages were surveyed across the country, along with the scoring of feather condition of 50 hens from each flock. Factors found to have an influence on greater feather damage seen in flocks included increasing age, having all brown-feathered hens, the practice of midnight feeding, and hens not having access to a scratching area or additional foraging material. These results support existing evidence that feather damage is the result of multiple factors, with genetics and foraging opportunity being some of the most important. Further research is needed to test the effectiveness of related intervention strategies.

**Abstract:**

Feather pecking is a continuous welfare challenge in the housing of egg-laying hens. Canada is currently making the transition from conventional cages to alternative housing systems. However, feather damage (FD) among laying hens due to feather pecking remains a welfare concern. An explorative approach was taken to assess bird, housing, and management associations with FD in Canadian laying hens housed in alternative systems. A questionnaire focused on housing and management practices was administered to 122 laying farms across Canada in autumn of 2017 (response rate of 52.5%), yielding information on a subset of 26 flocks housed in furnished cages. Additionally, a three-point feather cover scoring system was developed to estimate the prevalence of FD. Farmers assessed FD by sampling 50 birds per flock. Linear regression modeling was applied to explain FD as a function of 6 variables (out of an available 54). Of the 6 modeled variables, “increased age”, “brown feather colour”, “midnight feeding”, and “no scratch area” were associated with higher levels of FD at farm level (R^2^ = 0.77). The results indicated that FD resulting from feather pecking is a multifactorial problem, and supported existing evidence that FD increases as birds age. These results also suggested that “feather colour”, “midnight feeding”, and “access to (or lack of) a scratch area or additional substrate” play a role in FD prevalence in furnished cages.

## 1. Introduction

Today’s egg-laying hens face a multitude of welfare challenges, one of the most prominent being that of feather pecking (FP), which is experienced across all types of modern housing systems [1,2,3]. FP is a behaviour where hens peck, pull [4], or pluck at [5], and sometimes eat the feathers of their conspecifics [6,7], causing feather damage (FD), including feather loss, typically on the back/rump, vent, and tail area [8,9,10]. In addition to increased risk of abrasion and infection due to exposed areas of skin, loss of feather cover results in difficulty maintaining body temperature [11], and body balance [12], and can lead to mortality due to cannibalism of denuded areas [8]. FD thus significantly reduces bird welfare as well as increases economic losses for producers due to increased flock mortality and feed consumption, and reduced egg production [10,13,14]. The act of FP is considered to be a form of redirected foraging behaviour, where the feathers of other birds become a substrate of interest resulting in FD [15,16]. This behaviour is triggered by multiple factors including elements of housing design and environment, where stress and frustration can result from nesting, perching, dustbathing, and foraging needs not being met, in combination with factors such as rearing conditions, diet composition, and bird strain [17]. Genetic differences in particular can have significant impact on propensity to feather peck due to FP heritability [18], as well as level of fearfulness and thus the ability to cope with stressors that may trigger FP [19,20]. 

As of 2017, approximately 77% of Canadian laying hens are housed in conventional cages [21]. Use of alternative housing systems, such as furnished cages, single-tier floor systems, multi-tier aviaries, and free-range systems, which allow birds to express more natural behaviours, is, however, becoming more common in egg production in Canada [22] and in other countries [23,24]. In Australia, for example, the 2018 market share of free-range eggs by volume has now surpassed that of cage eggs at 45.4% and 44.0%, respectively [23]. The European Union even discontinued the use of conventional cages as of 2012 [24]. Canada is now following suit by transitioning out of conventional cage housing and into furnished cage and non-cage systems; a goal to be reached by July 1st, 2036 [25]. 

FP behaviour and FD outcomes can pose a greater risk in these alternative systems where a pecking bird has access to a larger number of pecking victims [26], although these housing systems can provide welfare benefits such as increased space allowance, nesting areas, and opportunities for dustbathing and foraging that conventional cages do not [25,27]. Birds in furnished cages are typically housed in groups of 10–100 birds [25] and >1000 birds in non-cage systems [2]. In these larger groups, FP can be transmitted through social learning and thus lead to sizeable FP outbreaks [28]. With the advent of new housing practices in Canada, identifying which farming factors contribute the most to poor feather cover and subsequently how to prevent and manage it, is especially needed at this time. 

Much research to determine influencing factors of FP and to quantify poor plumage condition has been done—primarily in Europe. Both experimental and epidemiological studies, with a focus on non-cage systems, have yielded genetics, stocking density, group size, rearing factors (e.g., perching, foraging, and dustbathing opportunities), floor type, feed, and light intensity, as main factors that impact FP behaviour and FD [29,30,31,32,33,34,35]. Comparatively little is known about FP and FD and its risk factors in a North American context, especially in furnished cages. Currently, furnished cages are increasingly implemented in Canada as one of the alternative housing systems for laying hens and are seen by egg farmers as a valuable compromise to conventional cages. 

The goal of this study was to report on putative risk factors for FD, as an indicator of FP behaviour, observed in furnished caged flocks in a Canadian setting as part of a larger project. The specific objectives were (i) to identify and quantify associations between FD and reported risk factors in the areas of management, environment, and genetics in laying hen flocks in housing systems alternative to conventional cages, (ii) to provide farmers with strategies to prevent/control FD in such systems, as well as (iii) to provide the industry with tools to assess and monitor feather cover damage.

## 2. Materials and Methods 

This study was part of a larger epidemiological project to investigate FD in laying hens kept in alternative housing systems on Canadian farms [22]. The study was conducted as described in Decina et al. [36], who also presents results for non-caged flocks. In brief, egg farmers were asked to 1) assess FD prevalence in their flock using a visual FD scoring system and 2) complete a comprehensive questionnaire based on housing features and management practices in a national survey. This data was used to 3) identify farm characteristics and practices associated with FD using regression modelling.

### 2.1. Development of the Feather Damage Scoring System

FD was assessed by farmers using a visual scoring system, which had been specifically designed for this project [36]. The system involved a three-point scale from 0 to 2 according to severity (Table 1) to allow scoring of both extremes of good and poor feather cover along with a more intermediate feather cover. A visual assessment of the back/rump area (the back region spanning from the shoulder to the base of the tail) was used to avoid the need for capture and handling, thus prioritizing user-friendliness and time efficiency. Visual scoring has been previously validated as an effective scoring method compared to capture and handling [37,38]. Additionally, FP is typically targeted at the back/rump area and it is less likely that damage in this area is caused by other conditions, such as abrasion from the system [17,29]. 

Farmers were asked to sample 50 birds selected evenly across all sections of the barn [32,39,40]. Scoring was performed once when farmers received their participation materials, with no specification for flock age. Farmers did not receive any formal training for the scoring system, however, the system was kept simple and detailed instructions with schematics to illustrate how to select birds, a feather cover scoring guide with full colour photographs of the scoring scales for white and brown birds, and recording sheets were provided. Farmers received a 10-dollar gift card for a popular Canadian coffee shop as incentive and thanks for participation.

### 2.2. Development of the Layer Questionnaire 

A questionnaire for laying hen farmers was adapted from Lambton et al. [41], which similarly looked at FP in laying hens in alternative systems and its associations with management and environmental factors. The questionnaire was adapted to comprise primarily housing and management-based questions specific to current Canadian practices and standards [25], using the expertise of the research team and feedback from federal and provincial egg boards. The main subsections of the questionnaire are outlined in Table 2. The questionnaire consisted of open-ended and closed questions with multiple answer options, and versions in both English and French were available. Both the questionnaire and the scoring instructions were pilot tested at Arkell Poultry Research Station (University of Guelph) as well as local farms representing alternative systems.

### 2.3. Questionnaire Distribution

In order to reach as many egg farmers as possible, questionnaire packages were provided to participants in both hard copy form via mail-out, and electronically via Qualtrics® online survey software (Qualtrics, Provo, UT, USA) [42]. Questionnaire package distribution was coordinated through the egg boards within each province to help maintain participant privacy using unique numeric codes on all documents. Documents included in the package were: (1) a laying hen questionnaire, (2) a feather cover damage scoring guide, (3) two feather cover damage scoring sheets—one for white hens and one for brown hens, (4) a cover letter outlining the study with a consent form, and (5) a return-addressed envelope for the return of written responses. 

Distribution of the survey began on October 3, 2017, and data was collected through to December 31, 2017. Reminders were sent out by the provincial egg boards 2–4 weeks post initial distribution and once more two weeks before data collection ceased. This study was approved by the University of Guelph Research Ethics Board (REB17-06-010). 

### 2.4. Statistical Methods 

FD prevalence was estimated as the percentage of sampled birds with back scores of 1 or 2 on each farm. Data obtained from the farmer questionnaires were used to determine factors associated with the prevalence of FD within a flock. All statistical analyses were performed using R version 3.4.3 “Kite-Eating Tree” [43] in combination with RStudio [44]. 

#### 2.4.1. Model Building

Data collected via the questionnaire was entered using double manual entry and checked for errors. Variables with excessive missing values (> 50% of responses missing) or with insufficient variation (e.g., a binary variable with a proportion of responses approximately > 0.85) were excluded from further investigation. Response categories for several variables underwent retrospective collapsing to remove unused and infrequent categories. After this screening, a total of 54 variables remained and were included in univariable analysis. Variables with some evidence of association to the outcome in a univariable analysis, i.e., reached the criterion of *p* ≤ 0.25, or were considered biologically relevant, were retained for further investigation. Continuous variables were checked for collinearity using Spearman’s rank-correlations. Associations between categorical variables were assessed with χ^2^-tests. Strong associations were deemed an indication for redundant variables and only one predictor variable was retained in the modelling process. Finally, 24 remaining predictor variables for FD prevalence were included in multivariable analysis using a mixed linear regression model with a forward variable selection approach. Variables that were significant (*p* ≤ 0.05) and/or contributed to a high adjusted R^2^ comprised the final model. Relevant interactions between retained predictor variables were tested. The variable for flock age was centered at 40 weeks in an effort to allow for a more intuitive interpretation of FD prevalence at this age. 

#### 2.4.2. Diagnostic Procedures

Model diagnostics were carried out to assess normality of residuals using a QQ-plot [45]. Homogeneity of variance was also evaluated graphically with a scatterplot of standardized residuals against fitted values. Collinearity was assessed using the Variance Inflation Factor (VIF). The presence of outliers was checked using a boxplot of model residuals, and the absence of influential data points was checked using Cook’s distance. 

## 3. Results

### 3.1. Response Rate

As a part of a larger cross-sectional study, a total of 122 questionnaire packages were distributed to laying hen farms where birds were not housed in conventional cages [22]. The number of packages returned totaled 64 (response rate of 52.5%), providing information for 65 flocks of which 26 flocks were housed in furnished cages (40.0%).

### 3.2. General Flock Information 

Flock size, flock age in weeks, and FD prevalence for these 26 flocks are presented in Table 3. Birds in all flocks were beak-trimmed at the hatchery (day 1) with an infrared laser. Most flocks had white-feathered birds (76.9%), while 23.1% of flocks were brown-feathered. Twenty-three farmers provided information on the breed of their flock showing that almost half (43.5%) were of Lohmann breed, while others included Bovans (4.3%), Dekalb (34.8%), Hy-line (4.3%), and ISA (13.0%). A detailed description of study flocks and housing and management practices on these farms is presented in van Staaveren et al. (2018). 

### 3.3. Univariable Analysis of Factors in Furnished Cages 

Housing and management factors associated at a liberal significance level (α = 0.25) with FD in furnished cage systems at the univariable level of analysis included the following: amount of farmer experience in years, age of the flock in weeks, stocking density defined as the number of hens per cage, feather colour, bird condition on arrival at the laying facility, inspection route, frequency of feeder running, and whether midnight feeding was used (Table 4). 

While the following variables did not meet the inclusion criterion of *p* ≤ 0.25, they were retained for multivariable model building due to their previously established connections with feather cover condition and/or their biological importance. These variables included: cage space allowance, provision of a scratch area and scratch substrate, rearing factors such as flock origin (same flock or multiple combined) and matching of barn conditions, length of daily inspections, use of a health plan, feed-related factors such as feed structure, diet changes, and provision of insoluble fibre, light type and intensity, and frequency of manure belt running (Table 4). 

### 3.4. Linear Regression Analysis for Furnished Cage Flocks 

The final linear regression model included six variables: “flock age (weeks)”, “feather colour”, “feed structure”, “frequency of feeder running”, “midnight feeding”, and “scratch substrate”. These factors accounted for approximately 77% of the variation in FD between flocks. Increasing “age”, brown “feather colour”, and “midnight feeding”, were found to be associated with higher levels of FD at the 5% significance level. “Frequency of feeder running” and no “scratch substrate” had a tendency of association with higher FD, while “mashed feed structure” was not found to be associated with FD, though did contribute to better explain the variation in FD between flocks (Table 5). 

## 4. Discussion

This study sought to assess associations between management, environmental, and genetic factors and FD outcomes in laying hen flocks housed in furnished cage systems in Canada. Findings indicate that on average, approximately 22% (95%CI: 10.4–33.4%) of the birds within these flocks exhibit some form of FD, either moderate or severe, when farmers perform assessments themselves. The factors found to have an influence on FD in Canadian flocks included older “age”, use of “brown-feathered birds”, and abnormal lighting cycle through “midnight feeding practices”. Lack of “scratch substrate” and “frequency of feeder running” tended to be associated with increased FD. 

Commentary on how the prevalence of FD found in the current study compares to the existing literature is somewhat difficult since little large-scale epidemiological investigation of FD has been done in commercial furnished cage flocks. Additionally, many studies in which plumage condition is assessed in such flocks report findings only in terms of mortality figures or average feather scores, rather than proportion of the flock affected, as reported here. Of the studies that have reported the proportion of FD, Sherwin et al. (2010) found that after comparison of 4 different UK housing systems, 24.9% of birds were affected by FD after scores were recorded at 30 and 70 weeks of age among 6 furnished cage flocks. Elson and Croxall [46], who similarly compared welfare between cage and non-cage systems in European flocks, found that all furnished cage flocks in the study had less than 25% of birds with naked back areas at 35 weeks of age, but by 60 weeks some flocks in large group furnished cages had percentages that exceeded 25%. The finding of 21.9% of birds with moderate to severe FD up to approximately 44 weeks of age in the current study is generally in line with those of the previous studies. It is likely, however, that the prevalence here is underestimated due to the wide age range of participating flocks where young flocks newly brought into lay may skew toward a lower prevalence if FP has not yet become apparent. FP behaviour and resultant FD have consistently been shown to increase as birds age [29,41,47], a finding corroborated here with a strong positive association between FD and age (0.71% increase in FP per week of age, *p* <0.001). Had the flocks of the current study been sampled at a uniform middle to late age like the previously mentioned studies, when FD would be apparent in at-risk flocks, prevalence may have been even higher. It is also important to note that farmers were responsible for scoring their own flocks for logistic reasons and that the accuracy of farmer assessment was not validated. The simplicity of the scoring system and visual instructions were thought to increase accuracy, and input from commercial farmers and provincial egg boards was sought before the start of the study. However, the self-assessment by farmers may contribute to potential underestimation of FD prevalence due to social desirability bias and the use of a novel system.

The greatest magnitude of effect on FD found in the present study’s furnished caged flocks was that of feather colour (*p* = 0.0017). Specifically, flocks with brown-feathered birds predicted 35% more FD than that would be found in white-feathered flocks. This finding is considered here as an indicator of genetic differences between breeds and/or strains. As specific breed information was not always provided by respondents, there was not enough data to reliably assess impact of breed as its own variable. Feather colour was instead used as a close proxy. The literature provides some limited evidence as to whether FD occurs more often in certain strains of brown- or white-feathered birds. De Haas et al. (2014) found that FD was found on more body areas and more injurious pecking behaviour occurred in ISA Brown hens compared to Dekalb White. Similarly, Yamak and Sarica (2012) observed significantly more plumage deterioration in brown compared to white layers. Contrastingly, Uitdehaag et al. [48] found that White Leghorn birds showed more FD than Rhode Island Red birds, and also exhibited more fearfulness (a factor involved in FP behaviour [49,50]). However, these findings were from studies using non-cage systems and battery cages, and therefore may not directly apply to furnished cage flocks. 

An alternative explanation for the marked difference in observed plumage condition between feather colour in this study could be simply that of observer bias. Brown birds tend to have an under-layer of white feathers that become more visible as the brown top layer is removed or gets damaged, thus FD, in general, may be more easily observed or perceived as damage from afar by a scorer for brown birds compared to white birds. This colour pattern may also affect birds’ own perception of an attractive pecking substrate as birds have been shown to feather peck in response to contrast of light and dark, see for example the studies by Keeling et al. [51], where white birds with brown pigmentation were more vulnerable to FP than all-white birds, and by Bright [52] who found that Oakham Blue birds with white plumage had less FD due to FP than black or grey birds. Interestingly, the study by McAdie and Keeling (2000), which looked at the effect of feather manipulation on FP and cannibalism in brown birds, found that birds with damaged feather cover received significantly more severe feather pecks than those with undamaged feathers. Their feather manipulations through trimming revealed the feathers’ light-colored bases, also suggesting that color contrast could play a role in damaged feathers being an attractive FP stimulus in brown birds. 

The practice of midnight feeding, where the dark phase of the lighting cycle is interrupted for a period of 1–2 hours to encourage hens to eat at this time, is used to either promote general growth or as a way for birds to take in more calcium for egg formation [53]. This practice was found to have a strong, positive association with FD in furnished cage flocks, estimating an increase in FD by 24% (*p* = 0.0232). This practice is no longer allowed in Europe [54], but is still in use in North America and is viewed positively from a production standpoint, especially for use in summer months or hot climates [55]. Midnight feeding has not been well-explored from a welfare perspective and thus little is known regarding its possible negative effects on bird behaviour. In humans, however, the literature surrounding night shift and rotating shift work’s disruption of circadian rhythms suggests a negative impact on health. Numerous studies, especially in healthcare and hospital workers, have documented negative effects on mental health and behaviour such as increased symptoms of anxiety and depression [56,57], and increased irritability, tension, and anger [58]. It may be suggested that the added burden of interrupted sleep could exacerbate existing behavioural problems in hens and thus may be a plausible contributor to poor plumage condition.

It is also worth considering that midnight feeding may contribute to FD in one other aspect: birds at rest are more vulnerable FP victims for active birds. When midnight feeding is practiced it creates an environment where there is a mix of resting and active birds, likely increasing the chance of resting birds becoming victims of active birds performing FP during that time. This is similar to what is known about the use of dark brooders for rearing chicks. Dark brooders, which are curtained, box-like structures that provide conductive heat, allow birds to rest and stay warm in a dark environment, thus simulating the role of a brooding hen [59]. Farms that do not use dark brooders, but rather use whole house heaters, typically leave chicks exposed to continuous light in their early days of life [59]. Research, both experimentally [60] and on commercial farms [59], has determined that the use of dark brooders reduces FP during rearing and into lay. It is believed that dark brooders separate active and inactive chicks, thus allowing birds to rest undisturbed and avoid pecking from active birds. 

Lastly, having no scratch substrate present in furnished cages tended to be associated with FD (*p* = 0.0987). The largest effect was contributed by having no scratch area present in the cage at all, estimating an increase in FD of approximately 18% compared to flocks with access to a scratch area with substrate present. A scratch area, typically a plastic mat that may or may not be textured, allows birds to simulate foraging and dustbathing behaviours. This result is in accordance with much of the existing literature, as the importance of opportunities for foraging and dustbathing in FP prevention and good plumage condition has been well-documented [13,61,62,63]. Research suggests the additional provision of substrate improves the body integrity and plumage coverage of hens [64]. One possible reason why lack of substrate did not have a stronger effect may be that excreta accumulation on the scratch pads functioned as a reasonable foraging substrate. In an experimental investigation of laying hen behaviour in response to clean versus excreta-covered scratch pads, Pokharel et al. [65] demonstrated that hens displayed a preference for foraging on pads covered in excreta and visited them more frequently over the clean option. Additionally, hens have been found to voluntarily consume the excreta of other hens even in the presence of an excreta-free feed source, suggesting a role for excreta as foraging material [66] if no appropriate foraging material is available. This finding in the current study may alternatively be an indicator that the presence of a scratch area at all is what makes the largest difference in amount of FD, more than that of additional litter in a furnished cage environment. More likely, the sample of flocks that had access to substrate was not large enough to detect a difference between groups.

Though frequency of feeder running tended to be associated with higher FD, the magnitude of effect was very small and its impact does not have a clear explanation at this time. Therefore, this factor was not further considered. 

It is important to note that this was an exploratory study, and thus the *p*-Values exhibited should be considered exploratory *p*-Values [67]. Additionally, no age restriction was imposed on participating flocks. Therefore, the factors investigated here may not have yet reflected their impact on plumage condition at the time of feather cover assessment for young flocks.

## 5. Conclusions

For the first time in Canada, this study estimated the prevalence of FD on farms housing laying hens in furnished cages, revealing that on average, 22% of birds display moderate or severe FD due to FP. It is evident that FD currently poses a problem for Canadian farmers and for those transitioning into furnished cage housing in the coming years. The findings here suggest that providing birds with the opportunity to forage using scratch areas in cages continues to be an important element in preventing FD in laying flocks, and that midnight feeding practices may induce FP stress. Brown birds may be more at risk for FD in furnished cage housing, however further research is needed to elucidate specific strain differences. The value of undisturbed rest for hens should not be underestimated and should be studied further in North American settings, as well as whether white or brown-feathered birds are more suited to furnished cage housing. 

Much research has been done in exclusively non-cage, free-range, and/or organic flocks in Europe and Australia. Comparisons of those findings to those found in the present study are not always directly applicable. Consequently, further investigation into furnished cage management impacts on FD is needed under commercial conditions and in diverse regions. 

## Figures and Tables

**Table 1:** The scoring system used by farmers on-site to evaluate the feather condition and amount of feather damage present in their flock. Body areas scored were limited to the back/rump.

Score	Body Condition
0	Intact feather cover, no or slight wear, only single feathers missing
1	Damaged feathers (worn/deformed) or bald patch visible ≤ a $2 coin
2	At least one bald patch visible that is > a $2 coin

**Table 2:** A summary of the housing and management information about a farmer’s current laying hen flock collected through the self-administered questionnaire.

**General Information**	Date Years of farming experience Province Farm size
**Flock Information**	Hatchery & rearing farm birds came from Date of placement Age of placement Current flock age Flock size at placement & current size
**Housing Features**	No. of cage tiers and rows Manufacturer & model Age of system Stocking density Perches (availability, height, space) Cage scratch area (availability, type, foraging material, cleaning) Nests (availability, type, location) Drinker & feeder type Enrichment (types, age of access, motivation for use)
**Bird Characteristics**	Feather colour Breed
**Rearing and Placement**	Visitation of pullet flock Home-rearing vs. supplier, integration of flocks yes/no Pullet housing system Beak trimming (yes/no, age, method, length) Condition on arrival Matching of environmental conditions
**Flock Health**	Inspection (frequency, duration, no. of workers, route, observations) Feather pecking (if it had been observed, body area, at what age, any management changes in response) Flock behaviour in response to workers Biosecurity measures Vaccination & instances of illness Mortality (percentage & main causes)
**Diet**	Feed structure, supplier, availability, supplements Feeding frequency & special practices (midnight feeding) Diet changes System breakdowns
**Lighting**	Type, hours of light, intensity Dawn/dusk period (yes/no) & method
**Air quality**	Type of ventilation Temperature, humidity, ammonia concentration, dust levels Manure removal frequency
**Productivity**	Age at start of lay No. of eggs collected per day, percentage of floor eggs Performance compared to breed standards Current & peak production figures

**Table 3:** Description of 26 laying hen flocks housed in furnished cages by average age, flock size and prevalence of feather damage (FD).

	N	Mean (SD)	Median (Range)
Flock age (wks)	26	43.6 (15.77)	43.5 (21–69)
Flock size	26	15,212 (9587.6)	13,006.0 (4371–47,721)
FD prevalence (%)	1300 *	21.9 (28.44)	6.0 (0–94)

* Total number of birds scored for FD (26 × 50).

**Table 4:** Housing and management factors (*p* ≤ 0.25) associated with the presence of feather damage in furnished cage laying flocks at the univariable analysis level.

Explanatory Variable	N (%)	Coefficient	*p*-Value
Farmer experience			
≤ 10 years	12 (46.2)	Referent	
More than 10 years	14 (53.8)	−15.31	0.1762
Flock age (weeks)	26 (100.0)	1.09	0.0010
Birds all from same rearing flock			
Yes	21 (80.8)	Referent	
No	5 (19.2)	−7.33	0.6145
Feather colour			
White	20 (76.9)	Referent	
Brown	6 (23.1)	36.50	0.0035
No. of hens/cage	26 (100.0)	0.40	0.1204
Cage space allowance (cm^2^)	26 (100.0)	0.04	0.4319
Scratch area			
Yes	14 (53.8)	Referent	
No	12 (46.2)	0.76	0.9474
Scratch Substrate			
Yes	8 (30.8)	Referent	
No	6 (23.1)	1.92	0.9057
No scratch area	12 (46.2)	1.58	0.9079
Matched housing type^+^			
Yes	3 (11.5)	Referent	
No	23 (88.5)	−17.42	0.3284
Matching of conditions*			
Yes	16 (61.5)	Referent	
No	10 (38.5)	0.78	0.9477
Manure belt frequency			
3-7x per week	3 (12.0)	Referent	
2x per week	12 (48.0)	26.17	0.1770
1x per week	10 (40)	21.13	0.2810
Light type			
LED	19 (73.1)	Referent	
No LED	7 (26.9)	−11.62	0.3660
Light intensity			
≤ 10 lux	11 (55.0)		
> 10 lux	9 (45.0)	5.98	0.6769
Feed structure			
Mash	18 (69.2)	Referent	
No Mash	8 (30.8)	−12.53	0.3096
No. of diet changes			
≤ 1 change	7 (28.0)	Referent	
2–3 changes	7 (28.0)	−0.86	0.9560
≥ 4 changes	11 (44)	14.99	0.2960
Gradual diet changes (yes/no/no change)			
Yes—gradual change	19 (73.1)	Referent	
No—immediate change	4 (15.4)	−22.37	0.1655
No diet change	3 (11.5)	−0.04	0.9984
Feeder running frequency (/day)	26 (100.0)	5.18	0.0387
Midnight feeding			
Yes	4 (15.4)	Referent	
No	22 (84.6)	−27.27	0.0771
Insoluble fibre in diet			
Yes	6 (25.0)	Referent	
No	18 (75.0)	−8.22	0.5602
No. of workers performing daily inspection			
1 worker	11 (42.3)	−11.84	0.3037
>1 worker	15 (57.7)	Referent	
Length of daily inspections			
< 45 mins	13 (50.0)	0.46	0.9680
≥ 45 mins	13 (50.0)	Referent	
Varied inspection route			
Yes	15 (57.7)		
No	11 (42.3)	13.69	0.2328
Injury/illness on arrival at laying barn			
Yes	4 (15.4)	Referent	
No	22 (84.6)	25.32	0.1024
Flock health plan in place			
Yes	6 (23.1)	Referent	
No	20 (76.9)	11.60	0.3919

^+^ Housing system type in which birds were kept during rear was the same used during lay * Whether conditions in the laying barn match those in which birds were kept during rear in terms of litter and perch availability, nutrition measures, and environmental aspects such as light and temperature

**Table 5:** Linear regression model of factors associated with feather damage prevalence in laying hen flocks housed in furnished cages (α = 0.05, adjusted R^2^ = 0.678, *p* < 0.001, N = 26).

Variable	Coefficient	SE	*p*-Value
Intercept	46.43	15.909	
Flock age (centered at 40w)	0.71	0.228	<0.001
Feather colour			0.0017
White	Referent		
Brown	34.60	9.039	
Feeder running frequency	2.45	1.540	0.0522
Midnight feeding			0.0232
Yes	24.39	9.202	
No	Referent		
Feed Structure			0.1872
Mash	13.20	7.697	
Pellets, grains or crumbs	Referent		
Scratch Substrate			0.0987
Yes	Referent		
No	14.16	9.079	
No scratch area	17.65	7.878	
